# The Determinant of DNA Repair Pathway Choices in Ionising Radiation-Induced DNA Double-Strand Breaks

**DOI:** 10.1155/2020/4834965

**Published:** 2020-08-25

**Authors:** Lei Zhao, Chengyu Bao, Yuxuan Shang, Xinye He, Chiyuan Ma, Xiaohua Lei, Dong Mi, Yeqing Sun

**Affiliations:** ^1^Institute of Environmental Systems Biology, College of Environmental Science and Engineering, Dalian Maritime University, Dalian, 116026 Liaoning, China; ^2^State Key Laboratory of Stem Cell and Reproductive Biology, Institute of Zoology, Chinese Academy of Sciences, Beijing, China; ^3^College of Science, Dalian Maritime University, Dalian, Liaoning, China

## Abstract

Ionising radiation- (IR-) induced DNA double-strand breaks (DSBs) are considered to be the deleterious DNA lesions that pose a serious threat to genomic stability. The major DNA repair pathways, including classical nonhomologous end joining, homologous recombination, single-strand annealing, and alternative end joining, play critical roles in countering and eliciting IR-induced DSBs to ensure genome integrity. If the IR-induced DNA DSBs are not repaired correctly, the residual or incorrectly repaired DSBs can result in genomic instability that is associated with certain human diseases. Although many efforts have been made in investigating the major mechanisms of IR-induced DNA DSB repair, it is still unclear what determines the choices of IR-induced DNA DSB repair pathways. In this review, we discuss how the mechanisms of IR-induced DSB repair pathway choices can operate in irradiated cells. We first briefly describe the main mechanisms of the major DNA DSB repair pathways and the related key repair proteins. Based on our understanding of the characteristics of IR-induced DNA DSBs and the regulatory mechanisms of DSB repair pathways in irradiated cells and recent advances in this field, We then highlight the main factors and associated challenges to determine the IR-induced DSB repair pathway choices. We conclude that the type and distribution of IR-induced DSBs, chromatin state, DNA-end structure, and DNA-end resection are the main determinants of the choice of the IR-induced DNA DSB repair pathway.

## 1. Introduction

Ionising radiation (IR), such as X- or *γ*-rays from medical radiation treatments, high-energy charged (HZE) particles from cosmic radiation, is an unavoidable risk factor to endanger human health [1–4]. IR can attack DNA and produce a variety of DNA lesions, mainly including DNA double-strand breaks (DSBs), DNA single-strand breaks (SSBs), mismatches, modified bases, and abasic sites, which are associated with various kinds of human diseases [5]. Among these DNA lesions, DSBs are considered to be the most deleterious DNA lesions, which are the major threats to genome integrity and stability, and the main factors to determine cellular fate (to survive, to carcinogenesis, or to die) after IR exposures [6, 7]. The evolutionarily conserved DNA repair pathways play critical roles in countering and eliciting IR-induced DSBs to ensure genome integrity and maintain genome stability [8, 9]. If these DNA lesions are not correctly repaired, residual or unrepaired DSBs can lead to the loss of genetic material and cell death, and especially incorrectly repaired DSBs can cause inappropriate end-joining and rearrangement events that may result in gene mutations, chromosome aberrations, cell transformation, carcinogenesis, etc. Therefore, the precise DSB repair is essential to reduce the IR-induced health risks. In contrast, genetic disruptions in the DNA repair pathways can cause genomic instability and enhance IR-induced health risks, especially carcinogenesis risks [10, 11].

Given the importance of DNA repair pathways in processing IR-induced DNA DSBs and reducing IR-induced health risks, many studies have been conducted to identify the critical proteins that recognise, transduce, and repair IR-induced DNA DSBs and to understand the complicated DNA DSB repair mechanisms in irradiated cells [12]. Some other studies have attempted to find the crucial gene transcriptions [13], noncoding RNAs [14], and posttranslational modifications (e.g., methylation, acetylation, and neddylation) [15] that probably implicated in the regulation of the DNA DSB repair pathways. Also, recent evidence shows that DNA repair pathways are expected to be highly evolutionarily conserved between different species [8, 9]. The conserved features of DNA repair pathways may facilitate interdisciplinary studies to expand the identification of undiscovered human DNA repair molecules for improving the new insights of the DNA repair mechanisms. Based on the conserved feature of DNA repair mechanisms, Nikitaki et al. [16] proposed an *in silico* approach to identify and rescue the candidate genes for the DNA repair pathways in plants and animals. We recently have also proposed a simplified *in silico* method of homologous comparison to investigate novel human genes implicated in DNA repair pathways. The results showed that many novel assembly proteins, transcription factors, and molecular chaperones were found to be involved in the IR-induced DNA repair pathways [17]. The innovative methods *in silico* modelling can be used to identify key DNA repair molecules and reveal IR-induced DNA DSB repair mechanisms.

There are four possible DNA repair pathways that have been employed concertedly by mammalian cells to repair DNA DSBs, such as classical nonhomologous end joining (c-NHEJ), homologous recombination (HR), single-strand annealing (SSA), and alternative end joining (alt-EJ) [18]. Accumulating evidence suggests that these repair pathways are not equal or alternative ways to handle DNA DSBs [19]. The disorder of the selections of DNA repair pathways can also cause the increases in DNA lesions, radiosensitivities, chromosomal translocations, carcinogenesis, and even other health risks [20, 21]. Indeed, a series of regulatory factors, such as the type and distribution of DNA lesions, local chromatin environment, and cell cycle phase, are employed to ensure that cells choose the suited DNA repair pathways [22–24]. Iliakis et al. [19] have proposed the hypothesis that the key factors in determining the engagements of DNA repair pathways are the degree of chromatin destabilisation generated by the DSBs. According to their opinions, indeed, different physical and biological factors have been found to determine the choices of DNA repair pathways mainly through the degree of chromatin destabilisation. However, despite all these, the understanding of the determinant of the IR-induced DNA DSB repair pathway choices is limited to date, and it is still unclear what determines the choices of major DNA repair pathways in response to IR-induced DNA DSBs.

In this review, we discuss how the mechanisms of IR-induced DSB repair pathway choices can operate in irradiated cells. We first begin with a description of the main characteristics of the major DNA repair pathways and the critical DNA repair proteins that dominate the repair of IR-induced DNA DSBs. We then mainly discuss the emerging understanding of the critical regulatory mechanisms of IR-induced DSB repair pathway choices. The overarching goal in this review is to summarize and highlight the main factors that probably determine the choices of IR-induced DSB repair pathways from different perspectives, which is of great significance for the mechanistic investigations of DNA repair pathways and consequential choices, the assessments of health risks, and even the developments of radioprotective or radiomitigative drugs after IR exposures.

## 2. Overview of IR-Induced DNA DSB Repair Pathways

**Figure 1:**
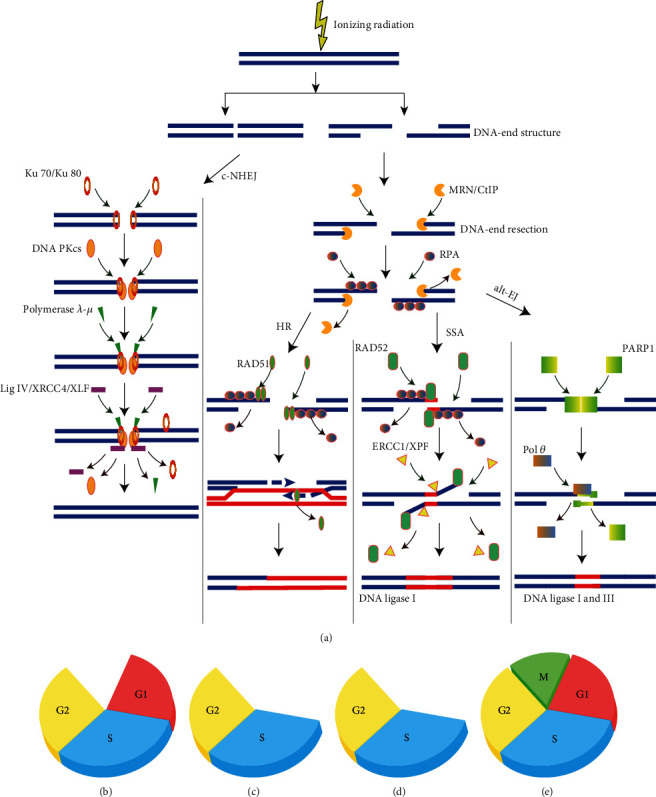
(a) Major repair pathways for DNA double-strand breaks (DSBs) generated by ionising radiation (IR). When IR-induced DNA DSBs have blunt double-strand DNA ends or contain short single-strand DNA ends, the classical nonhomologous end joining (c-NHEJ) is initiated by the binding of the Ku70/80 heterodimer followed by the recruitment of DNA-PKcs and polymerase. When DNA resection occurs, the pathways of homologous recombination (HR), alternative end joining (alt-EJ), and single-strand annealing (SSA) can be activated to repair the IR-induced DNA DSBs by the recruitments of different proteins. (b–e) The major repair pathways ((b) c-NHEJ, (c) HR, (d) SSA, and (e) alt-EJ) for processing IR-induced DNA DSBs have a distinct cell-cycle dependence.

As mentioned above, mammalian cells have probably employed four different DNA repair pathways to repair IR-induced DNA DSBs, each of which can repair DSBs using different mechanisms (Figure 1(a)). Among these pathways, c-NHEJ and HR are the two major pathways to repair IR-induced DSBs, while SSA and alt-EJ can repair the residual DSBs that are unable to be repaired by the c-NHEJ and HR [22]. Although many studies have been conducted for these pathways, the detailed molecular mechanisms of SSA and alt-EJ for repairing IR-induced DSBs are still not completely understood.

IR-induced DNA DSBs have blunt double-strand DNA ends or contain short single-strand DNA ends [18]. These kinds of IR-induced DNA DSBs can be repaired by c-NHEJ through joining two DNA ends in proximity to each other (Figure 1(a)). In contrast, IR-induced DSBs with long single-stranded DNA (ssDNA) tails and/or DNA-end resection that can be not repaired by c-NHEJ since long ssDNA tails can greatly reduce the affinity of Ku70/80, while which can be processed by the remaining pathways, HR, SSA, and alt-EJ (Figure 1(a)). The ssDNA tails in IR-induced DSBs can be stabilized by the invasions of replication protein A (RPA) [25]. The DNA-end resection can be carried out by the structure-specific nuclease (i.e., the MRE11/RAD50/NBS1 (MRN) complex) to generate “short-range resection”. And, the DNA-end resection can be implemented by the helicases and exonucleases, CtBP-interacting protein (CtIP), RPA, exonuclease 1 (EXO1), DNA replication helicase/nuclease 2 (DNA2), Bloom's syndrome (BLM) helicase, *etc*., to generate “long-range resection”.

In order to clarify the determinant factors of IR-induced DNA DSB repair pathway choices next, this section will briefly introduce the main characteristics of the four possible DNA repair pathways and the critical DNA repair proteins involved in these pathways as follow.

### 2.1. Classical Nonhomologous End Joining

The c-NHEJ is initiated by the binding of the heterodimer Ku70/80 to the blunt or short DSB ends induced by IR. The Ku70/80 binding at DSB ends may play roles in protecting the sequence of DNA ends from unnecessary resection. And then, the Ku70/80 mediates the recruitment of the DNA-dependent protein kinase catalytic subunit (DNA-PKcs), DNA ligase IV (LIG4), and the associated scaffolding factors XRCC4, XRCC4-like factor (XLF), and paralog of XRCC4, to ligate the broken DNA ends (Figure 1(a)) [26]. According to whether the ends can be ligated, additional end processing is needed to facilitate the c-NHEJ by several accessory factors, such as the nuclease Artemis and the specialized DNA polymerases *λ* and *μ* [27]. Also, other accessory factors, such as the Aprataxin and Aprataxin and PNK-like factor (APLF), can be involved in regulating the process of c-NHEJ. After IR exposures, histone H2AX can be phosphorylated at serine 139 through the recruitment of the above factors, resulting in forming of the discrete foci at DSB sites, which is the so-called ionising radiation-induced foci (IRIF) [28]. The *γ*-H2AX foci are one of the most sensitive biomarkers that can be used to reflect IR-induced DNA DSBs and plays a major role in the pathway of c-NHEJ.

In general, c-NHEJ represents the leading repair pathway to eliminate IR-induced DNA DSBs over all phases of the cell cycle except for M phase in irradiated mammalian cells (Figure 1(b)) [29]. Moreover, c-NHEJ is a simple, rapid, and highly efficient pathway to repair DSBs, which does not require a homologous sequence but depends on the blunt DNA-end structures[30]. Also, it should be noted that c-NHEJ frequently scavenges DNA ends by arbitrarily joining two ends that are very close in space. As a consequence, the c-NHEJ can result in some insertions or deletions in the genome, also known as translocations [31]. Due to the rapid speed for the repair of c-NHEJ and the fact that IR-induced DNA DSBs are usually very close to each other in space, c-NHEJ normally joins together the correct DNA ends, thus leading to low levels of translocations.

### 2.2. Homologous Recombination

The second major pathway of IR-induced DNA DSB repair is HR, which requires homologous sequences between sister chromatids. Also, HR is dependent on the DNA-end resection. When the range of DNA-end resection is a few thousand base pairs, the HR can be implemented for repairing through the invasion of DNA strand transferase RAD51 to achieve the RPA displacement (Figure 1(a)), which can promote sequence alignment between the homologous regions in sister chromatids to form the structure of Holliday junction (HJ). The RAD51 foci, as one kind of IRIF, can also be used as the biomarker for IR-induced DSBs and plays a central activity in the pathway of HR. During the process of HR, some DNA-end resection regulators, such as breast cancer type 2 susceptibility protein (BRCA2) and RAD51 paralogs (such as RAD51B, RAD51C, RAD51D, XRCC2, and XRCC3), can also be involved in facilitating the RPA displacement [32].

In general, HR is in most cases the error-free repair process that can faithfully restore the original DNA structure since it carries out recombination by using the homologous sister chromatid as a template even though HR also requires error-prone polymerases and can modify the forms of sequences through gene conversion and crossover [33]. Thus, under most conditions, HR can hardly cause chromosomal translocations or sequence modifications in the genome [34]. The complex processes involved in HR suggests that this pathway is relatively slower than c-NHEJ [35]. Moreover, HR is strongly dependent on the cyclin-dependent kinase (CDK) activity and therefore is largely restricted to the S and G2 phases of the cell cycle (Figure 1(c)) [36].

### 2.3. Single-Strand Annealing

SSA is also a DNA-end resection dependent pathway for DSB repair, which also requires the above-mentioned DNA-end resection proteins [37]. When the range of resection reaches a few hundred thousand base pairs, the SSA can be utilized to repair IR-induced DNA DSBs. Moreover, the repetitive sequence in the genome provides homologous regions for the engagement of SSA. However, few studies have analyzed the response of SSA in repairing IR-induced DNA DSBs.

Unlike the HR that relies on the invasion of RAD51, SSA requires the invasion of the strand accessory protein RAD52 to facilitate the displacement of RPA and to mediate the homology search between repeat regions (Figure 1(a)) [23]. Thus, RAD52 has an important role in the pathway of SSA. SSA mediates DSB end joining using the intervening sequences between the repeats in two single strands of DNA molecule. Although SSA is homology-dependent repair, SSA is still considered an error-prone repair, because the intervening sequence between the repeats can be deleted, thus resulting in the large deletions of sequences and the formation of many chromosomal translocations [38]. Similar to the HR, the SSA is also cell cycle dependent and has the potential activity during the S and G2 phases (Figure 1(d)) [19].

### 2.4. Alternative End Joining

Alt-EJ, also known as microhomology-mediated end joining (MMEJ), can join two IR-induced DNA ends together, which has similar principles as c-NHEJ [39]. Unlike the c-NHEJ, alt-EJ relies on the presence of microhomologous sequences within two limited DNA-end resections, which are typically ≥2 base pairs (bp) or 2~6 bp [40, 41].

In addition to the proteins mentioned above involved in the DNA-end resection, there are also some specific proteins implicated in the alt-EJ (Figure 1(a)). For example, poly(ADP-ribose) polymerase 1 (PARP-1) has been demonstrated to implement the displacement of Ku 70/80 or RPA, thus facilitating the pathway of alt-EJ [42]. Also, it should be emphasized that pol*θ* has both a C-terminal polymerase domain and an N-terminal helicase like domain and can further disclose the microhomologous DNA ends and promote the joint of the DNA ends [43]. That is to say that pol*θ* is essential for alt-EJ [41]. Furthermore, DNA ligases I and III can also be used in this pathway to promote the ligation of the DNA ends [18]. Although alt-EJ is active throughout the cell cycle, it also shows cell cycle dependence and has the maximum activity in the G2 phase (Figure 1(e)) [19].

Due to the relatively slow speed for the repair of alt-EJ and the fact that IR-induced DNA DSBs can diffuse away from the original position, alt-EJ subsequently increases the deletions and other sequence alterations and results in high levels of chromosomal translocations in the genome, which is much more extensive than those generated by c-NHEJ [23]. This mutagenic effect is generally considered to be less than those generated by the SSA.

## 3. The Determinant of IR-Induced DNA DSB Repair Pathway Choices

As described in the above discussion, the repair pathway choices are fundamental and essential to process IR-induced DNA DSBs, which are unique and not alternative ways to decide the cell fates [22]. According to the current advances of the regulation of the repair pathway choices in mammalian cells, in this section, we will systematically summarize and discuss the main possible factors and associated challenges to determine the IR-induced DSB repair pathway choices.

### 3.1. The Type and Distribution of IR-Induced DSBs Contribute to Repair Pathway Choices

**Figure 2:**
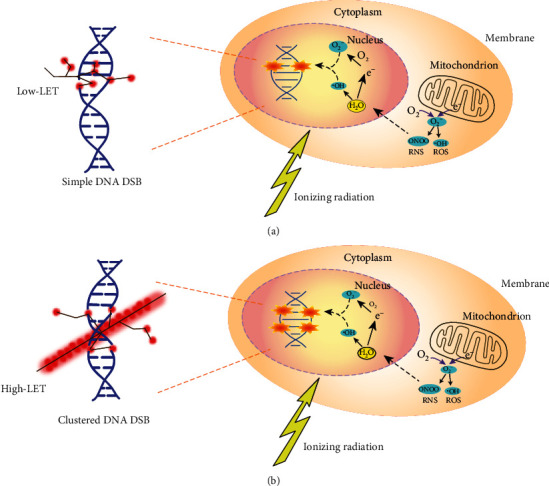
The direct and indirect effects of ionising radiation (IR) in cells. The schematic shows IR can lead to the DNA double-strand breaks (DSBs) directly by the induction of radiation energy deposition, or indirectly by the generation of reactive oxygen and nitrogen species (ROS and RNS). The direct effects are mainly determined by the radiation quality, i.e., low and high linear energy transfer (LET) can generate distinctive patterns of ionisation events on the structures of DNA molecules. When doses are the same, low-LET (a) and high-LET (b) radiation can generate different types and distributions of DNA DSBs. The IR-induced ROS and RNS are not only from the interaction of IR with water and molecules but also as a result of leakage of mitochondrial dysfunctions.

IR-induced DNA DSBs can occur in irradiated cells due to the direct effects through the energy deposition of IR [44–46], or the indirect effects through the generation of oxygen and nitrogen species (ROS and RNS) from the interaction of IR with water and molecules, and the leakage of mitochondrial dysfunctions [47] (Figure 2). Recently, studies in the field indicate that IR-induced DNA DSBs are mainly determined by radiation quality [48, 49]. One important parameter for depicting the radiation quality is linear energy transfer (LET), which describes the amount of energy deposition (or ionisations) generated by IR per unit of particle-track length [50]. When doses are the same, low- and high-LET radiation can generate different types and distributions of DNA DSBs. For example, low-LET radiation (such as X-rays and *γ*-rays) deposits its energy uniformly within cells. It generates primary simple DNA DSB lesions (e.g., isolated DSBs (iDSBs)), which are randomly distributed in the cell nucleus (Figure 2(a)). The simple DNA DSB lesions refer to a single and individual DNA DSB induced within a chromatin loop. In contrast, high-LET radiation (such as alpha ions and heavy ions) produces high ionisation densities and deposits lots of energy in a small distance along the track of each particle and produces high levels of ROS and RNS. Through this mechanism, high-LET radiation increases the yield of DSBs and induces more complex or clustered DNA DSB lesions (e.g., clustered DSBs (cDSBs)), which have significant track structure characteristics (Figure 2(b)) [51, 52]. The clustered DSB lesions refer to two or more close DSBs within a chromatin loop, which can also be composed of both DSB and non-DSB lesions [53]. Also, low-LET radiation with a high dose can also lead to complex or clustered DNA DSBs damage [54].

**Figure 3:**
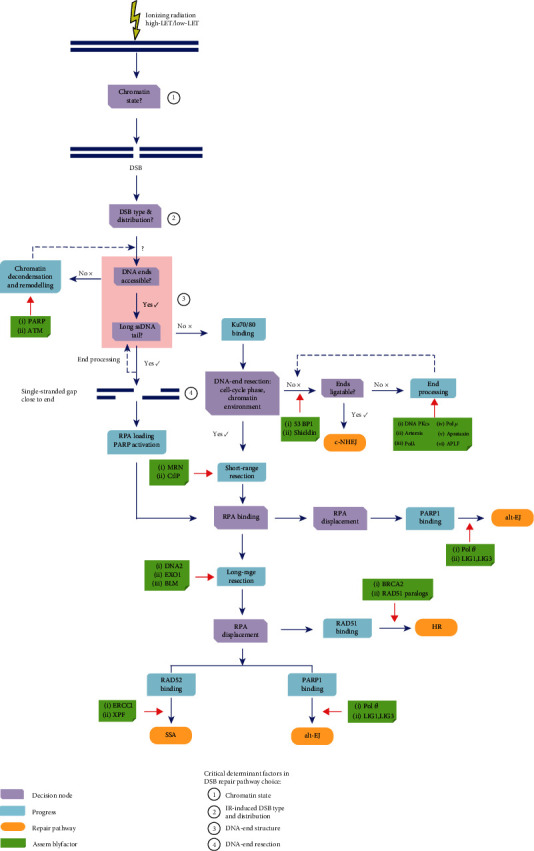
The choices of ionising radiation- (IR-) induced DNA repair pathways. The schematic shows that there are four key factors, including the chromatin state, the type and distribution of IR-induced DNA double-strand breaks (DSBs), the DNA-end structure, and the DNA-end resection, which can determine the repair pathway choices. Many critical proteins that are directly or indirectly involved in the above processes also play a critical role in determining the pathway choices. The more detailed discussions can be found in the main text.

According to the review of Sridharan et al. [54], the choices of IR-induced DNA DSB repair pathways may primarily depend on radiation quality. This is mainly due to the fact that different radiation qualities can result in different complexity of DNA lesions, such as simple DNA DSB lesions and clustered DNA DSB lesions, and thereby may trigger different repair pathways. That is to say that the types and distributions of IR-induced DNA DSBs can determine the DSB repair pathway choices (Figure 3).

In general, the iDSBs induced by low-LET radiation can be mainly repaired by the pathways of c-NHEJ and HR, while the cDSBs generated by high-LET radiation are difficult to repair [55]. For example, a recent study provides the evidence that there are distinct spatial structures of key DSB repair factors, such as *γ*-H2AX, tumor suppressor p53-binding protein 1 (53BP1), and RAD51, in the IRIF in Hela cells after high- or low-LET radiation exposures [56], which suggest that different types and distributions of IR-induced DSBs may elicit distinct repair pathways. The study of Okayasu et al. shows that the cDSBs induced by high-LET radiation may markedly depend on c-NHEJ [57]. Also, the experimental investigation of Sridharan et al. showed that Artemis, as a key assembled-protein in the c-NHEJ, is involved the repair of the clustered DSBs induced by high-LET radiation, which supports that the c-NHEJ has important roles in the repair of cDSBs generated by high-LET radiation [58]. However, Wang et al. have also found that the complex DSBs containing short DNA fragments induced by high-LET radiation can also inhibit the c-NHEJ, which is possible due to this kind of DNA DSB lesions making it difficult for the heterodimer Ku70/80 to load onto the DNA ends and results in less efficient c-NHEJ-mediated DSB repair [59]. Also, Zafar et al. [60] investigated the contribution of DSB repair pathways in repairing DSBs induced by high-energy iron ions. They found that some key HR proteins in the process of DNA-end resection and DNA strand invasion are also involved in repairing the complex DSBs generated by high-LET radiation. In addition, they found that the assembly factors in the HR-deficient rodent cells are sensitive to high-LET radiation, resulting in enhanced induction of mutation and chromosome aberration. Therefore, the above evidence indicates that the type and distribution of IR-induced DSBs can be a leading factor to contribute to repair pathway choices. However, to our knowledge, the contributions of c-NHEJ and HR to repair cDSBs generated by high-LET radiation are still not well understood [54]. Moreover, the residual simple and clustered DSBs induced by IR with different radiation qualities can be repaired by the pathways of alt-EJ and SSA [19], while the detailed processes of the two pathways in response to high- and low-LET radiation is still unclear.

### 3.2. The Roles of Chromatin State in IR-Induced DSB Repair Pathway Choices

The chromatin state can change the forms of IR-induced DSBs and therefore affect the consequences of repair processing, thus indicating that the chromatin state may also have important roles in influencing the repair pathway choices (Figure 3) [61]. In the euchromatin, the genome is active for DNA replication and transcription. Therefore, the DSBs generated by IR in this region are likely to be handled by the extensive DNA-end resection [20]. Several studies have also indicated that the IR-induced DSBs in the euchromatin are mainly repaired by c-NHEJ and HR [62, 63]. Unlike the euchromatin, the IR-induced DSBs in the region of heterochromatin disfavour the pathway of HR and prefer the pathway of alt-EJ, while remaining the activity of c-NHEJ unaltered [64]. Also, some studies found that to allow the pathway of HR for repairing IR-induced DSBs in the heterochromatin, it requires to increase the levels of the poly(ADP-ribose) polymerase (PARP) and ataxia telangiectasia mutated protein (ATM) and other assembly factors to disassemble the chromatin [65, 66]. Moreover, the repair kinetics of IR-induced DSBs in the heterochromatin is significantly slower than that of euchromatin [20], which is consistent with the above conclusions.

Taken together, we infer that the IR-induced DSBs produced in different chromatin states may lead to different consequences of DSB repair pathways. Additionally, the PARP and ATM, which may promote chromatin decondensation and remodelling, are part of early response signals to IR-induced DSBs and have important roles in determining the repair pathway choices (Figure 3). Indeed, in addition to the above mechanism, transcriptions, noncoding RNAs, and epigenetic modifications can also be implicated in determining the roles of chromatin state in pathway choices, which need be considered in the further studies.

### 3.3. The Roles of DNA-End Structure in IR-Induced DSB Repair Pathway Choices

The DNA-end structure is also an important factor to determine the initial IR-induced DSB repair pathway choices (Figure 3) [20, 22]. As mentioned above, if the IR-induced DNA DSB ends are blunt, the heterodimer Ku70/80 will be prone to bind to the DNA ends to protect the DNA-end structure and facilitate the c-NHEJ. However, if the IR-induced DNA DSB ends contain long ssDNA tails or single-stranded gaps close to DNA ends, it can block the Ku70/80 binding to the DNA ends since Ku70/80 binds weakly to this kind of DNA-end structure, and directly lead to the RPA loading and PARP activation, which can result in the choices of the DNA-end resection dependent DSB repair pathways. According to the above descriptions, the DNA-end resection dependent DSB repair pathways include the HR, SSA, and alt-EJ [19]. However, if the additional end processing occurs in this kind of DNA-end structure, it may also evade end recognition by Ku70/80, which can promote the c-NHEJ.

In general, IR can generate DNA DSBs with blunt ends, or with some modified DNA ends, such as long ssDNA tails or single-stranded gaps [67, 32, 53]. Also, high-LET radiation can produce the clustered DSBs, especially the presence of the non-DSB lesions around the DSB site [54]. Thus, the IR-induced clustered DSBs with sharp DNA ends or single-stranded gaps close to DNA ends can hinder the Ku70/80 binding to the ends of DSBs, which limits the pathway of c-NHEJ and favours the DNA-end resection-dependent DSB repair pathways. It could probably explain why, sometimes, the complex DSBs induced by high-LET radiation can inhibit the c-NHEJ and promote the HR [59]. However, the study of Povirk et al. also indicated that the Artemis nuclease can be used to process this kind of DNA-end structure induced by IR and can thereby promote the c-NHEJ [68].

Overall, the spatial structure of the IR-induced DNA DSB ends has important roles in determining the IR-induced DSB repair pathway choices (Figure 3). The IR-induced DNA-end structure primarily depends on radiation quality, while the quantitative relationship between LET and DNA-end structure is unclear.

### 3.4. The Roles of DNA-End Resection in IR-Induced DSB Repair Pathway Choices

IR-induced DSBs can also be further processed through the DNA-end resection. The processes of DNA-end resection can remove the heterodimer Ku70/80 from the DNA DSB ends and activate the alternative pathways (such as HR, SSA, and alt-EJ) for DSB processing. It indicates that the DNA-end resection plays a critical and essential role in determining the IR-induced DSB repair pathway choices. And, according to recent advances, there are many factors that can affect the process of DNA-end resection [22, 23]. We will illustrate the roles of DNA-end resection in IR-induced DSB repair pathway choices from two aspects.

#### 3.4.1. The Roles of Chromatin Environment in IR-Induced DSB Repair Pathway Choices through Regulating DNA-End Resection

The key assembly factors in chromatin environments that block DNA-end resection can make Ku70/80 retention at DNA ends, which can promote the c-NHEJ. Conversely, the factors in chromatin environments that contribute to DNA-end resection can make the displacement of Ku70/80 and facilitate the DNA-end resection-dependent DSB repair pathways (Figure 3). For example, 53BP1 has one of the important roles in determining the DSB repair pathway choices through restricting the DSB-end resection [69]. 53BP1 can be recruited to the IR-induced DSB ends and form IRIF around DSBs, thereby promoting the chromatin compaction, blocking the DNA nucleases access to the DSB ends [70], and limiting the length of DSB end resection [71]. Therefore, 53BP1 can be considered to have an important role in promoting the pathway of c-NHEJ. The number of 53BP1 foci can also be used as another surrogate marker for DSBs and the corresponding irradiation dose, and the formation and disappearance of IR-induced 53BP1 foci are similar to those of *γ*-H2AX foci [72]. Recent studies reported that the Shieldin complex has similar repair functions as 53BP1, which can suppress the DNA-end resection, convert ssDNA tails into blunt ends, and facilitate the c-NHEJ [73, 74]. Conversely, BRCA2 and RAD51 paralogs, as the antagonistic of the 53BP1 and Shieldin complex, can overcome the barrier against DNA-end resection after IR exposures and promote the RAD51 loading [75] and further lead to the activation of HR.

Furthermore, the length of the DNA-end resection is most likely to be the main reason for influencing the IR-induced DSB repair pathway choices (Figure 3) [22]. If the range of resection is less than 20 bp, also known as “short-range resection,” the pathway of alt-EJ will have the opportunity to be activated by an important step of the PARP1 binding. If the range of resection is relative long (about a few thousand bp), known as “long-range resection,” the pathway of HR will be mainly required for repairing DSBs by the recruitment of DNA strand transferase RAD51. In addition, if the range of resection is a few hundred thousand bp, the SSA pathway can also be chosen opportunistically for repairing the residual DSBs through the invasion of RAD52 nucleofilament. Lastly, if DNA ends are the long-range resection and have the microhomologous repeat region, the alt-EJ pathway will be possibly chosen for repairing through the competitive binding of PARP1.

Indeed, many different kinds of accessory factors, such as DNA repair proteins or complex and posttranslational modifications, can contribute to the regulation of the length of DNA-end resection (Figure 3) [76, 77]. These factors can be changed by IR exposures and thereby influence the decision of IR-induced DSB repair pathway choices [19, 23, 78]. For example, MRN, CtIP, BRCA1, DNA2, EXO1, and BLM, as key DNA repair proteins or complex, are implicated in regulating the length of the DNA-end resection [19], which is very important in controlling the DSB repair pathway choices. Most of these proteins acted as the key helicase and nuclease to generate ssDNA with end resection, which is stabilized by the invasion of RPA [19]. The inhibition of any proteins mentioned above can inhibit the pathways of HR and SSA for repairing the IR-induced DSBs but allows the pathway of c-NHEJ [24].

Moreover, ATM or ATR, known as two major kinases that are differentially activated after low- and high-LET radiation [79], can recruit and phosphorylate the members of the MRN complex (MRE11, RAD50, and NBS1) and subsequently cause the phosphorylation of other key DNA repair proteins, such as CtIP, BRCA1, EXO1, and BLM. ATM or ATR can significantly enhance the efficiency of DNA-end resection and promote the related DSB repair pathways [80]. Therefore, ATM or ATR can be also considered the crucial regulators of the DNA-end resection and determine IR-induced DSB repair pathway choices. In addition, some posttranslational modifications have also been implicated in the DNA-end resection by regulating the activities of either ATM or ATR [23].

In addition to the proteins that are directly or indirectly involved in the DNA-end resection, there are other key proteins that could influence the IR-induced DSB repair pathway choices (Figure 3) [22, 23]. For example, some proteins that implicated in the displacement of RPA are capable of determining the choices of the IR-induced DSB repair pathways [22]. Specifically, the RAD51 binding to displace the RPA on the ssDNA can promote the pathway of HR, the RAD52 binding to displace the RPA can lead to the pathway of SSA, and the PARP1 and pol*θ* binding to displace the RPA can facilitate the pathway of alt-EJ [43].

Therefore, according to the above discussions, we can conclude that DNA-end resection is considered the main factor to control the IR-induced DSB repair pathway choices. A series of critical proteins, such as 53BP1, BRCA2, RAD51 paralogs, ATM, ATR, MRN, CtIP, BRCA1, DNA2, EXO1, BLM, RPA, PARP1, and pol*θ*, can affect IR-induced DSB repair pathway choices through regulating the length of the DNA-end resection. However, it is not very clear what the difference between low- and high-LET radiation responses of these proteins in the chromatin environment is. More radiation biological experiments and biophysical models should be used to clarify this issue.

#### 3.4.2. The Roles of the Cell Cycle in IR-Induced DSB Repair Pathway Choices through Regulating DNA-End Resection

In addition to the above factors that can affect DNA-end resection, cell cycle is also an important factor to regulate the process of DNA-end resection (Figure 3), which can also determine the choices of IR-induced DNA DSB repair pathways. That is to say that the phase of cell cycle has an important role in DSB repair pathway choices [36]. The supporting evidence is that c-NHEJ is required for the repair of IR-induced DSBs over all phases of cell cycle except for M phase (Figure 1(b)); alt-EJ is active for repairing IR-induced DSBs throughout the cell cycle and has the maximum activity in the G2 phase (Figure 1(e)). At the same time, HR and SSA are primarily utilized during S and G2 (Figures 1(c) and 1(d)). The main reason given for these dependencies is that the CDK activity can be increased significantly when cells enter the S and G2 phases. The CDK activity can activate the DNA repair proteins through phosphorylation to perform the DNA-end resection [81]. For example, the CDK-dependent CtIP and EXO1 phosphorylations can facilitate the DNA-end resection in the S/G2 phases, which significantly promotes the pathway of HR [82, 83]. On the other hand, the impairment of the CtIP and EXO1 phosphorylations can decrease the DNA-end resection and further enhance the pathway of c-NHEJ. That is, in the G1 phase, the CDK activity can be significantly reduced to limit the DNA-end resection and therefore favours c-NHEJ over DNA-end resection dependent repair pathways [77].

Experimental evidence has shown that IR can lead to the activation of DNA lesion-dependent cell cycle checkpoint controls and result in the cell cycle arrest in G1/S or G2/M [84]. The cell cycle arrest not only provides sufficient time for DNA repair but also changes the CDK activity [85], which can regulate the process of DNA-end resection. We can, therefore, conclude that the cell cycle can also determine the IR-induced DSB repair pathway choices through CDK activity affecting the DNA-end resection. Moreover, the effects of different LETs on cell cycle checkpoint controls should be paid more attentions for further studies, which may be a key factor that cannot be ignored in regulating the IR-induced DSB repair pathway choices.

## 4. Conclusion

Based on the current research advances in the characteristics of IR-induced DNA DSBs and the regulation of the repair pathway choices in irradiated cells, we have systematically summarized and discussed four key factors including the type and distribution of IR-induced DSBs, chromatin state, DNA-end structure, and DNA-end resection, to determine IR-induced DSB repair pathway choices. Additionally, we have also proposed some associated challenges for future studies, including (1) the contributions of c-NHEJ and HR in repairing cDSBs induced by high-LET radiation; (2) the roles and mechanisms of alt-EJ and SSA in repairing IR-induced DSBs; (3) the roles of transcriptions, noncoding RNAs, and epigenetic modifications in regulatingthe chromatin state and thereby determining IR-induced DSB repair pathway choices, and (4) the relationships between LET and initial DNA-end structure, the critical proteins involved in DNA-end resection, and cell cycle checkpoint controls.
